# Functional Analysis of Retinitis Pigmentosa 2 (RP2) Protein Reveals Variable Pathogenic Potential of Disease-Associated Missense Variants

**DOI:** 10.1371/journal.pone.0021379

**Published:** 2011-06-27

**Authors:** Suresh B. Patil, Toby W. Hurd, Amiya K. Ghosh, Carlos A. Murga-Zamalloa, Hemant Khanna

**Affiliations:** 1 Department of Ophthalmology, University of Massachusetts Medical School, Worcester, Massachusetts, United States of America; 2 Department of Ophthalmology and Visual Sciences, University of Michigan, Ann Arbor, Michigan, United States of America; 3 Department of Pediatrics and Communicable Diseases, University of Michigan, Ann Arbor, Michigan, United States of America; 4 Department of Pathology, University of Michigan, Ann Arbor, Michigan, United States of America; Indiana University, United States of America

## Abstract

Genetic mutations are frequently associated with diverse phenotypic consequences, which limits the interpretation of the consequence of a variation in patients. Mutations in the retinitis pigmentosa 2 (*RP2*) gene are associated with X-linked RP, which is a phenotypically heterogenic form of retinal degeneration. The purpose of this study was to assess the functional consequence of disease-associated mutations in the *RP2* gene using an in vivo assay. Morpholino-mediated depletion of *rp2* in zebrafish resulted in perturbations in photoreceptor development and microphthalmia (small eye). Ultrastructural and immunofluorescence analyses revealed defective photoreceptor outer segment development and lack of expression of photoreceptor-specific proteins. The retinopathy phenotype could be rescued by expressing the wild-type human RP2 protein. Notably, the tested RP2 mutants exhibited variable degrees of rescue of rod versus cone photoreceptor development as well as microphthalmia. Our results suggest that RP2 plays a key role in photoreceptor development and maintenance in zebrafish and that the clinical heterogeneity associated with RP2 mutations may, in part, result from its potentially distinct functional relevance in rod versus cone photoreceptors.

## Introduction

Recent advances in whole genome sequencing and exome capture techniques have exploded the field of identification of new disease in patients with genetic diseases [1], [2]. However, even patients with mutations in the same gene frequently exhibit immense clinical heterogeneity, ranging from early-onset disorders to relative less severe late onset disease. Such phenomena pose a challenge to computationally predict and interpret the effect of the mutation on the penetrance and severity of disease phenotypes.

Degeneration or dysfunction of photoreceptors is frequently associated with variable clinical presentation, likely due to their unique structure and metabolic demands [3]. Photoreceptors are polarized neurons with a distinct inner segment (IS) and photoreceptive outer segment (OS), linked by a narrow bridge-like microtubule-rich structure, called connecting cilium [3]. Proteins, such as rhodopsin, destined for outer segments are synthesized in the IS and are transported via trans-Golgi network to the base of cilium from where they are transported apically by microtubule-based motor assemblies [4]. Additionally, there is passive motor-independent bidirectional transport of some phototransduction proteins [5], [6], [7]. Owing to high degree of protein trafficking demands, dysfunction in protein synthesis, sorting or trafficking results in photoreceptor dysfunction and degenerative disorders [8].

Retinitis Pigmentosa (RP) represents one such disorder that exhibits both clinical and genetic heterogeneity in patients [9]. RP is characterized by progressive loss of rod and cone photoreceptors of the retina, resulting in night blindness followed by complete blindness [10], [11], [12]. To date, more than 200 RP-associated genes have been identified. RP is inherited in autosomal dominant, recessive as well as X-lined manner [13]. X-linked RP is one of the severe forms of RP, phenotypically characterized by onset of night blindness in the second decade of life that progresses into legal blindness by the age of 40 [14], [15]. There are six genetic loci and only two cloned genes for XLRP: retinitis Pigmentosa GTPase regulator and retinitis Pigmentosa 2 (RP2). While mutations in RPGR account for 70–80% of XLRP, RP2 mutations are known to occur in approximately 20% of XLRP patients [16], [17], [18].

The *RP2* gene (NM_006915.2)encodes a polypeptide of 350 amino acids [19]. Previous biochemical and cell biological studies have revealed a potential role for RP2 in maintaining Golgi cohesion and targeting of proteins to plasma membrane [20], [21], [22]. Some disease-associated mutations in RP2 abolish plasma membrane targeting of RP2 in cultured cells. The amino-terminal region (151 amino acids) of RP2 shares homology with tubulin-specific chaperone protein (TBCC) and also acts as a potential GTPase activating protein (GAP) for small GTPase ADP-ribosylation factor like-3 (ARL3) [23], [24], [25], [26]. The carboxyl-terminus of RP2 is homologous of nucleoside diphosphate kinase (NDK); however, the physiological function of this domain remains to be determined. We have recently shown that RP2 localizes to sensory cilia and interacts with polycystin-2, a protein involved in renal ciliary diseases [27].

We and others have reported the association of wide-spectrum of clinical phenotype in patients with RP2 mutations. These clinical features include severe to late-onset disease, typical RP, as well as macular dystrophy [18], [28], [29]. Disease causing mutations in RP2 are presented in the form of splice site, missense, nonsense and/or frameshift variations in the coding region. These mutations seem to affect distinct protein interactions, likely due to their effect on RP2 localization or interaction with other proteins. Hence, detailed analysis of effect of RP2 mutations on its function should provide insights into associated disease progression and pathogenesis.

It has been shown that analysis of zebrafish embryos provides an excellent platform to analyze in vivo effect of silencing of genes followed by disease progression and pathogenesis [30], [31]. Moreover, rescue of the phenotypes by human mRNA provides an analytical assay system to ascertain the effect of disease-associated mutations on the function of the protein, which in turn, can provide novel insights into associated patient phenotype. In the present study, we have utilized zebrafish as a platform to assess the pathogenic potential of selected RP-associated disease mutations in RP2. Our results indicate potentially distinct effects of RP2 mutation on cone versus rod photoreceptor dysfunction, the heterogeneity frequently observed in RP2 patients.

## Results

### Suppression of rp2 in zebrafish results in retinopathy

**Figure 1:**
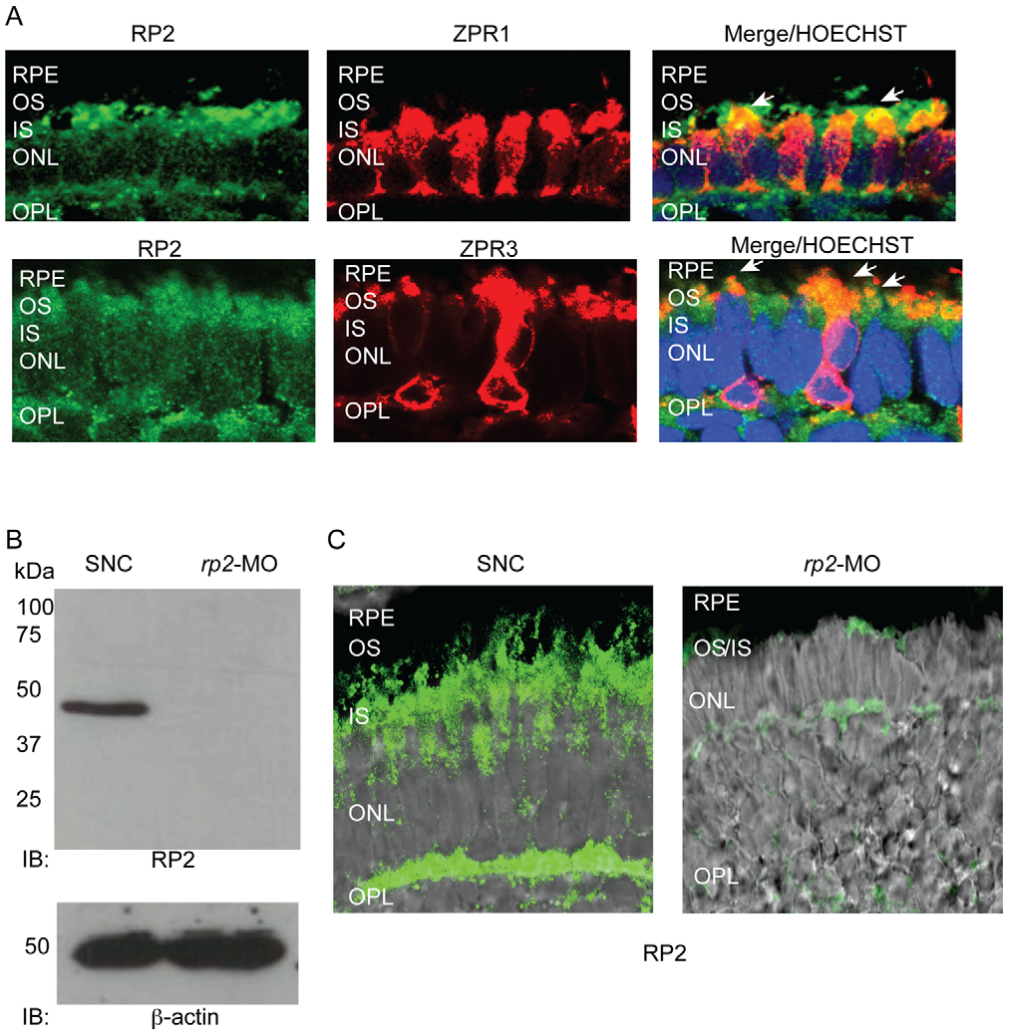
RP2 is expressed in zebrafish photoreceptors. **A**. Retinal cryosections from 4 dpf zebrafish embryos were analyzed by immunofluorescence using anti-Rp2 antibody (green). Photoreceptor markers, Zpr1 and Zpr3 (red) were used to stain cone and rod photoreceptors, respectively. Arrows in Merge indicate co-localization (yellow) of RP2 with both markers. Nuclei are marked with Hoechst (blue). RPE: retinal pigmented epithelium; OS: outer segment; IS: inner segment; ONL: outer nuclear layer; OPL: outer plexiform layer. **B**. Protein extracts from 4 dpf zebrafish embryos injected with standard negative control (SNC) morpholino (MO) or *rp2*-MO were analyzed by SDS-PAGE and immunoblotting using anti-RP2 antibody. The expected size band of 42 kDa was detected in the SNC-treated embryo extracts but not in the *rp2*-MO treated ones. Lower panel shows immunoblotting of same samples using anti-β actin antibody, as loading control. **C**. Immunofluorescence analysis of cryosections of SNC and rp2-mo treated embryo retina (4 dpf) was performed using anti-RP2 antibody (green). Merge with Nomarski image shows that the expression of RP2 was significantly down regulated in the *rp2*-MO treated embryos.

To gain insight into RP2 function, we first sought to determine the localization of RP2 in zebrafish photoreceptors. Using a previously reported anti-RP2 antibody [27], we found that RP2 is expressed in both rod and cone photoreceptors, as determined by staining with photoreceptor markers Zpr3 (rods) and Zpr1 (specific to cone surface epitope on red green double cones) antibodies (Figure 1A). These findings are consistent with the reported rod and cone degeneration phenotypes observed in patients with RP2 mutations. To ascertain the effect of knockdown of *rp2* function, we designed a translation blocking (AUG) morpholino against the zebrafish *rp2* gene (NM_213446.1) and injected into 2–4 cells stage wild-type (WT) zebrafish embryos [27]. We used 5 ng as an optimal concentration, based on a survival curve to derive the concentration of the MO to achieve maximal phenotype with minimal lethality [27] (data not shown). Knockdown of RP2 protein expression was validated by immunoblotting and immunohistochemistry (Figure 1B, C). RP2 protein expression can be detected in standard negative control (SNC) MO injected embryos but not in *rp2*-MO injected embryos (Figure 1B). Immunofluorescence analysis further corroborated these findings, as indicated by a lack of detection of RP2-associated signal in the zebrafish embryo retina in MO-injected fish (Figure 1C).

As RP2 mutations cause retinal degeneration in patients, we utilized retinal degeneration phenotype as a read out for the effect of silencing of the *rp2* gene in zebrafish. Our analysis revealed that suppression of *rp2* in zebrafish results in microphthalmia (small eye) (∼15% of defective embryos) and abnormal photoreceptor development in a majority (∼90%) of embryos, although retinal development seemed to proceed normally, at least up to 5 dpf. However, injection of higher doses of the *rp2*-MO resulted in defective retinal lamination (data not shown). Measurement of the eye diameters on the histological sections indicated approximately 32% reduction in size of defective embryo eyes compared to control embryos compared (1.10 mm±0.01 in control eyes and 0.74 mm±0.04 in defective embryo eyes; p<0.005) (Table 1).

**Table 1:** Diameters of the zebrafish eye.

Injection of embryos	Mean±S.E.
WT RP2	1.19±0.015
SNC	1.10±0.014
*rp2*-MO	0.74±0.046
*rp2*-MO+GFP	0.65±0.008
*rp2*-MO+RP2 GFP	1.13±0.023
*rp2*-MO+C86Y-GFP	0.67±0.015
*rp2*-MO+P95L -GFP	0.64±0.015
*rp2*-MO+C108G- GFP	0.85±0.037
*rp2*-MO+ R118H-GFP	1.00±0.027
*rp2*-MO+ E138G-GFP	1.10±0.024
*rp2*-MO+L188P-GFP	0.70±0.011
*rp2*-MO+ R282WGFP	0.65±0.022

**Figure 2:**
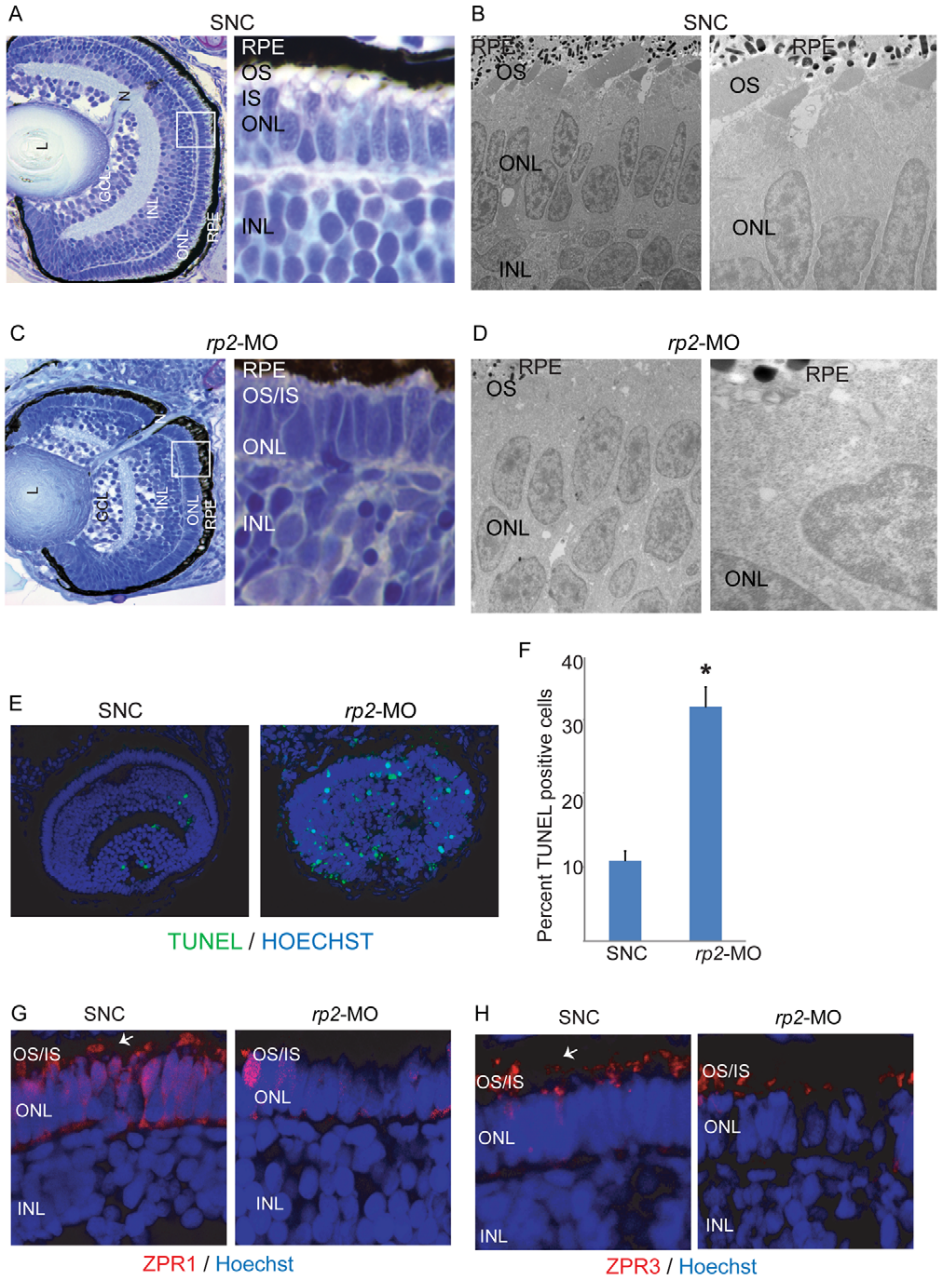
Silencing of *rp2* results in retinopathies in zebrafish. **A & C**. Histological analysis of zebrafish retina treated with SNC (A) or *rp2*-MO (**D**) was performed at 4 dpf. Insets show enlarged image of the area marked in white square. **B & D**. Electron microscopy analysis of SNC (**B**) and *rp2*-MO (**D**) treated embryo retinas shows defective OS development due to knockdown of rp2. Right panels are higher magnification images of the left panels. **E**. TUNEL staining of the indicated zebrafish retinas was performed using manufacturer's instructions. Green nuclei indicate pycknotic cells. The TUNEL staining was quantified and represented in panel **F**. *: p<0.001. **G, H**. SNC and *rp2*-MO treated embryo retina cryosections were stained with Zpr1 and Zpr3 antibodies (red; arrows). Nuclei were stained with Hoechst (blue). OS: outer segment; IS: inner segment; ONL: outer nuclear layer; INL: inner nuclear layer.

Histological analysis of zebrafish retinas at 4 days post fertilization (dpf) further revealed very few to no outer segment development in the defective embryos (Figure 2A, C). Disruption of OS development was further validated by electron microscopy analysis (Figure 2D). To test cell death, we performed TUNEL staining on control and defective embryo retinas at 3, 4, and 5 dpf. We found that, at 4 dpf, total number of TUNEL positive cells was statistically higher (38%) than control retinas (12%) (Figure 2E, F). Similar increase in TUNEL positive cells in defective embryos was observed at 3 and 5 dpf, indicating that silencing of *rp2* results in early developmental defects in zebrafish embryos (data not shown).

We next investigated the effect of depletion of *rp2* on the expression and localization of photoreceptor-specific proteins in the embryo retinas. To this end, we performed immunofluorescence analysis of control and *rp2*-MO treated 4 dpf embryo retina using Zpr1 and Zpr3 antibodies. As shown in Figure 2G, H, suppression of *rp2* results in a loss of both rod and cone photoreceptor specific staining pattern, which also indicates a defect in photoreceptor morphology (Figure 2G, H), consistent with electron microscopy results. Similar results were obtained when immunofluorescence was performed using anti-Rhodopsin (1D4; rod specific opsin) or peanut agglutinin (PNA; cone cell marker) (Figure S1). There seemed to be a consistently more pronounced effect of rp2 depletion on Zpr1 staining compared to Zpr3, which indicates a distinct role of RP2 in cone photoreceptor formation and maintenance.

### Human RP2-encoding mRNA rescues the *rp2*-MO associated phenotype

**Figure 3:**
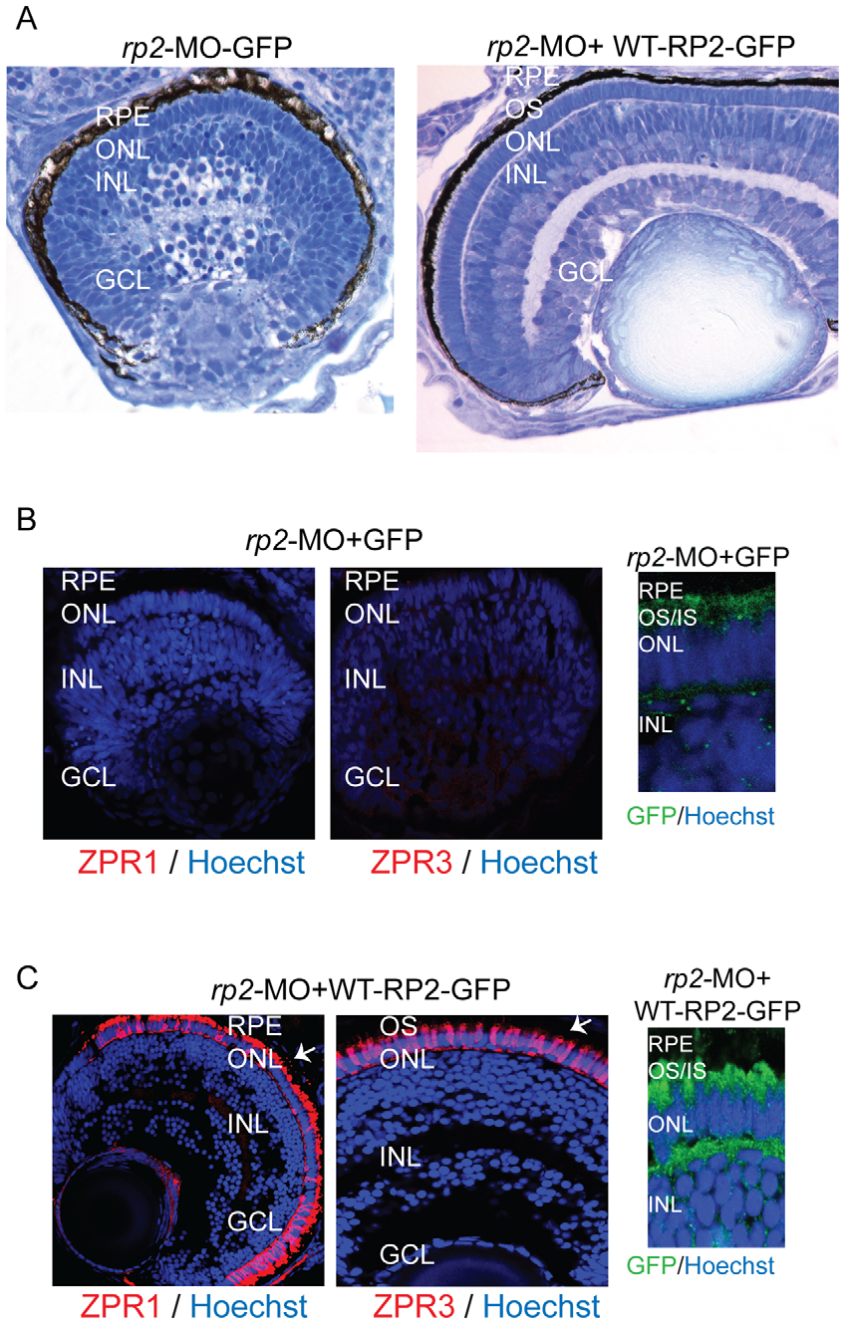
Human WT RP2 can rescue *rp2*-MO associated phenotype. **A**. Histological analysis of retinas from embryos co-injected with *rp2*-MO and mRNA encoding GFP (left panel; *rp2*-MO + GFP) or wild type (WT) RP2-GFP fusion protein (right panel; *rp2*-MO + RP2-GFP). OS: outer segment; IS: inner segment; ONL: outer nuclear layer; OPL: outer plexiform layer. **B** and **C**. Immunofluorescence analysis of the indicated embryo retinas was performed using Zpr1 or Zpr3 antibodies (red; arrows) or anti-GFP antibodies (green). Hoechst was used to stain nuclei (blue). OS: outer segment; IS: inner segment; ONL: outer nuclear layer; INL: inner nuclear layer; GCL: ganglion cell layer.

As human and zebrafish *RP2* genes are highly conserved [27], we hypothesized that human *RP2* mRNA can complement the loss of RP2 in zebrafish. To this end, we performed rescue experiments by co-injecting human RP2 mRNA with *rp2*-MO. The mRNA was engineered to encode RP2 protein fused to GFP (green fluorescent protein) at its carboxyl-terminus. We found that the human mRNA could efficiently rescue the retinopathy phenotype associated with suppression of *rp2*, whereas mRNA encoding GFP alone did not result in a rescue. Histological and immunofluorescence analyses revealed preservation of photoreceptor morphology and expression of rod and cone-specific proteins in the retina of embryos treated with mRNA encoding RP2-GFP fusion protein. As shown in Figure 3A, B, while the *rp2*-MO treated embryos injected with mRNA encoding GFP alone did not show OS development and expression of Zpr1 or Zpr3 (Figure 3B), injection of mRNA encoding RP2-GFP fusion protein resulted in the development of OS and expression of photoreceptor specific proteins (red signal in Figure 3A, C). Consistently, we detected expression of GFP or RP2-GFP proteins in the treated retinas (Figure 3B and C).

### Human RP2 mutations exhibit diverse pathogenic potential

As the human RP2 mRNA could rescue the *rp2*-MO associated retinopathy phenotype, we utilized the *rp2*-MO zebrafish as an assay system to investigate the pathogenic potential of selected nonsynonymous amino acid variations observed in patients. The rationale for selecting nonsynonymous variations is that prediction power of the effect of changes in the amino acid sequence on the three-dimensional structure of the protein is limited by the *in silico* algorithms. Additionally, if a protein possesses enzymatic activity, as is in this case, single amino acid variations can result in mild to adverse effect on the function depending upon the resultant affinity for the substrate or activity towards the substrate.

**Figure 4:**
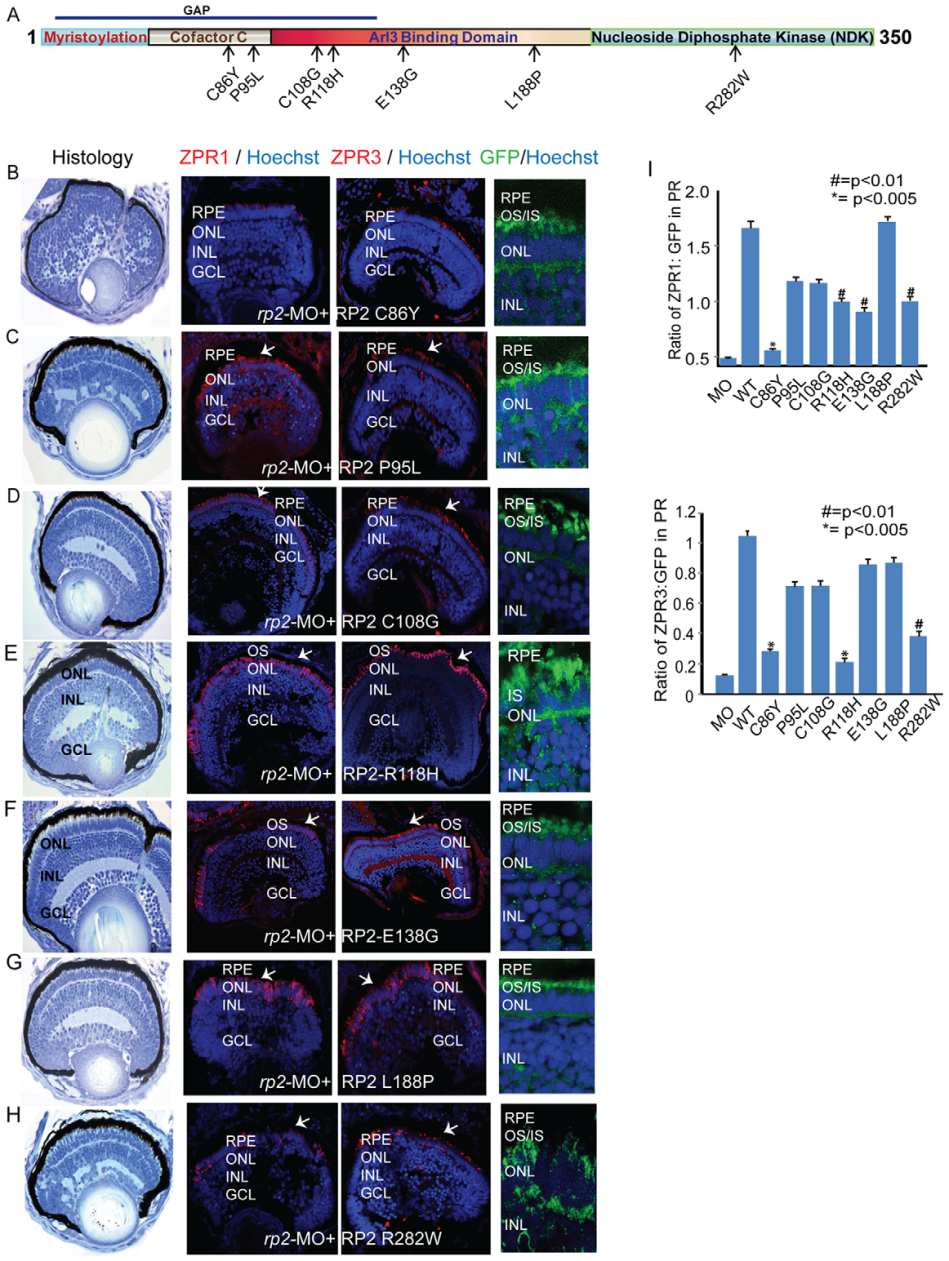
RP2 disease mutations exhibit diverse pathogenic potential. **A**. Schematic representation of the primary structure of the RP2 protein and predicted domains. Arrows indicate the mutations tested in this study. GAP: GTPase activating protein domain. **B–H**. Histological and immunofluorescence analyses (using ZPR1 or ZPR3 antibodies; red; arrows or anti-GFP; green) of retinas of embryos injected with *rp2*-MO and mRNA encoding indicated RP2 mutant proteins (fused to GFP) was performed as described in the Methods section. Nuclei are stained with Hoechst (blue). RPE: retinal pigmented epithelium; OS: outer segment; IS: inner segment; ONL: outer nuclear layer; INL: inner nuclear layer; GCL: ganglion cell layer. **I**. Quantitative analysis of ZPR1 and ZPR3 expression in the photoreceptor layer of the different embryos was performed and compared to expression of the WT or mutant RP2-GFP protein. The results are represented as ratio of Zpr1 or Zpr3 expression to the GFP expression in photoreceptors.

We selected seven nonsynonymous RP2 variations reported in patients that encompassed the GAP domain and the NDK-like domain (Figure 4A). As the proteins are expressed as a fusion with GFP, the expression levels of the different mutant proteins were compared to the extent of the rescue of Zpr protein expression achieved in the treated photoreceptors. We utilized three parameters to assess the pathogenicity of the RP2 mutants: (i) microphthalmia, (ii) histological analysis of retina, and (iii) expression of Zpr1 and Zpr3 in photoreceptors. Co-injection of mRNA encoding the selected RP2 variants with *rp2*-MO revealed a spectrum of phenotypic rescue. While *RP2* R118H and *RP2* E138G resulted in eye diameter that was comparable to that with WT RP2, other mutants did not seem to achieve a rescue of the microphthalmia in defective embryos (Table 1). Mutants *RP2* P95L, *RP2* C108G and *RP2* L188P although exhibited partial rescue of photoreceptor morphology, as observed by formation of photoreceptor OS, did not seem to completely complement the expression of Zpr1 and Zpr3 (Figure 4). On the other hand, mutants *RP2* C86Y, *RP2* R118H and *RP2* E138G revealed interesting observations. While the photoreceptor morphology and levels of Zpr protein expression achieved with the C86Y mutation were comparable to that obtained with GFP alone (Figure 4B, I), the R118H mutation exhibited statistically significant rescue of Zpr1 expression (∼50% compared to GFP alone) (Figure 4E, I). However, the R118H mutation behaved as a null allele in the context of rod photoreceptor-specific Zpr3 expression (Figure 4E, I). On the other hand, *RP2* E138G exhibited partial complementation of RP2 function in rod photoreceptors (Zpr3 expression) but not in cone photoreceptors (Figure 4F, I). One notable observation was the lack of rescue observed with the *RP2* R282W mutation (Figure 4H). This variation was reported as a polymorphism in one study. However, for potential reasons discussed later, our results suggest that this nonsynonymous variation renders the RP2 protein partially inactive.

## Discussion

The complexity associated with diverse clinical features of a disease due to mutations in a single gene has hampered our ability to predict the degree of severity of the phenotype as well as the cell types that are prone to dysfunction and degeneration. As RP2 is proposed to function as an activator of small GTPase ARL3 and may be involved in membrane trafficking, it is critical to examine the effects of mutations in photoreceptors. As photoreceptors are one of the highly metabolically active neurons and involve immense transport of proteins from the inner to the photosensory outer segments, even slight perturbations in protein trafficking machinery results in photoreceptor degeneration and blindness [32], [33], [34]. Our results indicate that involvement of rod versus cone photoreceptors in RP2-associated retinopathy depend upon the specific protein interactions and signaling pathways that are affected by a particular mutation in the *RP2* gene.

In this report, we have analyzed the functional consequence of mutations in RP2 on its function using in vivo approaches. The use of zebrafish as an experimental platform has distinct advantages, as it shares common developmental and morphological characteristics with higher vertebrates, including humans [35]. Additionally, the zebrafish model provides an excellent assay system to examine physiologically relevant phenotypes, including retinal development and degeneration [30], [36], [37]. These are particularly relevant to assessing the pathogenic potential of mutations that have a relatively low frequency of occurrence and are not amenable to traditional genotype-phenotype correlation studies. Our studies provide an assay system to functionally correlate the effect of disease-causing mutations of RP2 to disease progression. In our analysis, the RP2-C86Y mutant protein could not completely rescue the ZPR1 as well as ZPR3 expression, indicating a dysfunction of both cone and rod photoreceptors. On the other hand, a common disease-associated RP2 variant R118H exhibited partial rescue of Zpr1 staining but not of Zpr3. The R118H mutation results in reduced affinity of RP2 for ARL3 and is associated with severe photoreceptor degeneration in some patients [26], [29]. On the other hand, the E138G mutation seems to partly rescue the rod photoreceptor-associated phenotype. Recent studies showed that the residue Glu 138 is involved in its association with NSF, a protein involved in membrane trafficking [22]. It is however, possible that residues Arg118 and Glu138 are involved in additional ARL3- and NSF-independent roles, respectively. Further studies are needed to dissect such roles.

It was intriguing to note that the R282W variation revealed dysfunction of RP2 in both rod and cone photoreceptors. This is particularly surprising since R282W was initially identified as a polymorphism and was observed in control population albeit at low frequency [18]. We speculate that this mutation may be detrimental to RP2 function and affect its interaction or intracellular localization but does not, by itself, retain a pathogenic potential. Sequence alterations in other genes may be required for the complete penetrance and severity of the retinal degeneration phenotype. This hypothesis is supported by our previous report that a hypomorphic allele of RPGRIP1L, A229T, although affects protein function in zebrafish rescue assays, is frequently associated with retinal degeneration in patients with founder mutation in a different gene [37].

Notably, our data seem to be consistent with previously reported observations of pathogenicity of *RP2* mutations in patients. We found a spectrum of severity associated with RP2 mutations, as revealed by different degree of rescue of cone versus rod photoreceptor development. These results suggest that context-dependent function of RP2 in photoreceptors should be considered when analyzing disease pathogenesis. Moreover, our studies corroborate the clinical findings of RP2 patients that exhibit classic RP as well as macular atrophy starting at an early age [29]. We do acknowledge that the role of RP2 in photoreceptor development in zebrafish embryos may not extrapolate to human condition. This is because RP2 patients, based on clinical features and age of onset of disease, do not exhibit a photoreceptor developmental anomaly. In addition, there is a lack of evidence of any RP2 patients exhibiting extra-retinal phenotypes. Abnormal developmental defects observed in this study could be reminiscent of the zebrafish system wherein a multi-system developmental anomaly may depend upon the degree of knockdown of gene expression. The mutant RP2 proteins tested in this study were able to partly rescue the developmental phenotype observed due to knockdown of *rp2*. Thus, these RP2 mutations may be hypomorphic in nature. However, involvement of species-specific regulators of protein expression and function cannot be ruled out at this stage. Studies are underway to generate mammalian models of RP2 dysfunction that would be amenable to designing rational therapeutic approaches.

## Materials and Methods

### Ethics statement

All animal procedures were approved under the protocol A2221-10 titled “Retinal degeneration due to defective protein trafficking in photoreceptors” by our Institutional Animal Care and Use Committee of the University of Massachusetts Medical School, which operates under approval number A-3306-01 from the Association of Assessment and Accreditation of Laboratory Animal Care (AAALAC).

### Antibodies

Rabbit anti-RP2 antibody was generated by immunizing rabbits with full length recombinant human GST-RP2 and characterized for specificity, as described [27]. Mouse anti-acetylated α-tubulin and rabbit anti-actin antibodies were obtained from Sigma (Sigma Aldrich, St. Louis, MO). Alexa 564 was obtained from Invitrogen (Carlsbad, CA). Zpr1 and Zpr3 were purchased from www.Zfin.org. Rhodopsin 1D4 antibody was a kind gift of Dr. Robert S. Molday.

### Histology, immunofluorescence, and ultrastructural analyses

Histological analysis on zebrafish retina was performed on JB4 plastic sections as previously described [38]. Paraformaldehyde fixed embryo were analyzed for immunofluorescence as described earlier. Electron Microscopy was done using standard protocols. Briefly, zebrafish were fixed in perfused with 4% paraformaldehyde and 2.5% glutaraldehyde in PBS. The fish were treated with osmium tetroxide and dehydrated using graded ethanol series followed by treatment with propylene oxide and embedded in Epon-812 resin. Gold interference (50–80nM) sections mounted on grids were post stained uranyl acetate (5% in ethanol), and lead citrate (4% aqueous solution), washed with water and dried before imaging under Philips C10 TEM at 80 KV.

### Morpholino mediated knockdown and capped RNA synthesis

To knock down zebrafish RP2, we synthesized a translation blocking morpholino (Gene-Tools) (AUG-MO 5′<GGCCTGTCAGCATAA>3”) [27]. A standard mismatch morpholino (Gene tools) was used as negative control. To rescue the MO-induced phenotype, the zebrafish *RP2* gene was cloned into pEGFP-N1 (Clonetech) vector and mutations were created using QuickChange mutagenesis kit (Stratagene). Capped mRNA was synthesized *in vitro* with mMessenger mMachine T7 kit (Ambion).

### Zebrafish phenotype analysis

Zebrafish **(** *Danio rerio)* were reared and maintained as previously described [31]. Embryos were analyzed at 4 dpf for tail extension anomalies hydrocephaly, curved tails, edema, pericardial effusion and micro-ophthalmic phenotypes. The effect of various mutants mRNA was assessed as percent defective embryo compared to morpholino alone. Fluorescence intensity was analyzed on cryosections using Leica Application Suite and results were expressed as ratio of intensities for Zpr1 or Zpr3 to GFP.

## Supporting Information

Figure S1 **RP2 is expressed in both rod and cone photoreceptors.** Immunofluorescence analysis of 4 dpf zebrafish embryos injected SNC-MO and *rp2*-MO was performed using anti-rhodopsin (1D4) antibody or PNA (red). Nuclei are stained with DAPI (blue). RPE: retinal pigmented epithelium; OS: outer segment; IS: inner segment; ONL: outer nuclear layer; INL: inner nuclear layer; GCL: ganglion cell layer.
(TIF)
