# Supplementary figures and images for: Functional Analysis of Retinitis Pigmentosa 2 (RP2) Protein Reveals Variable Pathogenic Potential of Disease-Associated Missense Variants
Source: PLoS One. 2011 Jun 27;6(6):e21379. doi: 10.1371/journal.pone.0021379 (PMC3124502; doi:10.1371/journal.pone.0021379)

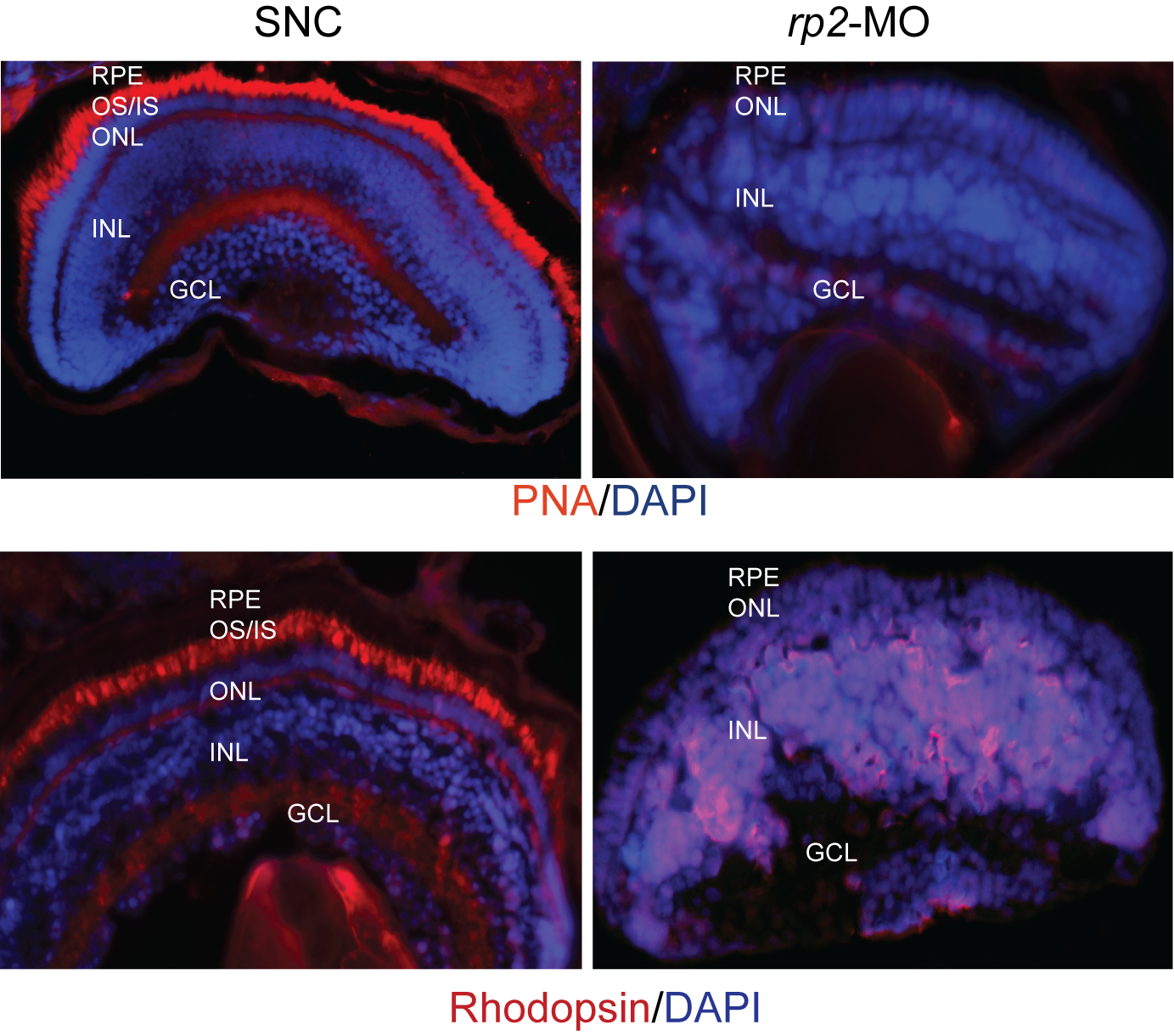

Supplement: Figure S1 — RP2 is expressed in both rod and cone photoreceptors. Immunofluorescence analysis of 4 dpf zebrafish embryos injected SNC-MO and rp2-MO was performed using anti-rhodopsin (1D4) antibody or PNA (red). Nuclei are stained with DAPI (blue). RPE: retinal pigmented epithelium; OS: outer segment; IS: inner segment; ONL: outer nuclear layer; INL: inner nuclear layer; GCL: ganglion cell layer. (TIF) [file pone.0021379.s001.tif]
